# Ultra-Translucent Zirconia Laminate Veneers: The Influence of Restoration Thickness and Stump Tooth-Shade

**DOI:** 10.3390/ma16083030

**Published:** 2023-04-11

**Authors:** Salwa Mekled, Salma Elwazeer, Carlos A. Jurado, James White, Faddy Faddoul, Kelvin I. Afrashtehfar, Nicholas G. Fischer

**Affiliations:** 1 Temple University Kornberg School of Dentistry, Philadelphia, PA 19140, USA; 2 Texas Tech University Health Sciences Center El Paso Woody L. Hunt School of Dental Medicine, El Paso, TX 79905, USA; 3 The University of Iowa College of Dentistry and Dental Clinics, Iowa City, IA 52242, USA; 4 Evidence-Based Practice Unit, Clinical Sciences Department, Ajman College of Dentistry, Ajman City P.O. Box 346, AE, United Arab Emirates; 5 Department of Reconstructive Dentistry and Gerodontology, School of Dental Medicine, University of Bern, 3010 Bern, BE, Switzerland; 6 Artificial Intelligence Research Center (AIRC), Ajman City P.O. Box 346, AE, United Arab Emirates; 7 Minnesota Dental Research Center for Biomaterials and Biomechanics, University of Minnesota School of Dentistry, Minneapolis, MN 55455, USA

**Keywords:** dentistry, prosthodontics, zirconia, shade, dental veneers, laminate veneers, color alteration, spectrophotometry, color mapping, color matching, aesthetic outcomes, CAD/CAM

## Abstract

This in vitro study aimed to evaluate the final shade of translucent zirconia laminate veneers with varying thicknesses over teeth with different shades. Seventy-five chairside computer-aided design/computer-aided manufacturing (CAD/CAM) shade A1 third-generation zirconia dental veneers, with thicknesses of 0.50 mm, 0.75 mm, and 1.00 mm, were placed on resin composite teeth with shades ranging from A1 to A4. The laminate veneers were divided into groups based on thickness and background shade. All restorations were evaluated with a color imaging spectrophotometer, to map the veneer surface from A1 to D4. Regardless of the thickness or background shade, all dental veneers showed color alteration from the original shade. Veneers with 0.5 mm thickness tended to display the B1 shade, while veneers with 0.75 mm and 1.0 mm thickness primarily exhibited the B2 shade. The thickness of the laminate veneer and background shade significantly modified the original shade of the zirconia veneer. One-way analysis of variance was performed and a Kruskal–Wallis test was used to determine the significance between the three veneer thicknesses groups. The results indicated that the thinner restorations showed higher values with the color imaging spectrophotometer, suggesting that thinner veneers may result in more consistent color matching. This study underscores the importance of carefully considering thickness and background shade when selecting zirconia laminate veneers, to ensure optimal color matching and overall aesthetic outcomes.

## 1. Introduction

Dental zirconia is a popular ceramic material, due to its aesthetic outcomes, biocompatibility, toughness, and low production cost [1,2,3,4,5]. Although zirconia was initially introduced as a core material, its use as a monolithic restorative material has become more common, due to concerns over chipping when layered [6,7]. A systematic review found that a zirconia core layered with another ceramic can have a chipping rate of 24% after 3 years of use, leading to the switch to monolithic applications [8]. Furthermore, the composition of dental zirconia has undergone significant changes over time [9,10]. First-generation zirconia was completely opaque and used only as a core ceramic. Second-generation zirconia (3Y-YZP) was modified by reducing the alumina content, resulting in some translucency and the ability to be used as a monolithic material, although it remains somewhat opaque. Third-generation zirconia (5Y-YZP) was modified by increasing the yttria content from 3% to 5% or more, resulting in considerably increased translucency [9,10,11,12].

To ensure the success of zirconia laminate veneers in terms of mechanical properties and esthetic demands, it is crucial to balance their translucency and color matching with natural teeth [13,14,15,16]. Although zirconia ceramics exhibit partial translucency, variations in light transmission have been reported, which can impact the aesthetic outcome [17,18,19]. Factors such as the substrate, cement, type of zirconia, sintering thickness, and aging can also affect the shade of zirconia restorations [20,21,22,23,24,25]. While increasing the thickness of the zirconia restoration can improve background masking properties, it reduces translucency [19,26]. Moreover, porosity, grain size, oxide additives, and light absorption during cement polymerization can influence the dispersion and absorption of light, thereby affecting the final shade of the restoration [27,28,29]. Thus, carefully considering the tooth shade, shade selection, and thickness of a zirconia restoration is necessary to obtain the desired aesthetic outcome.

Translucent zirconia (5Y-TZP) has emerged as a potential material for aesthetic veneer restorations, but few case reports in the literature have demonstrated desirable final shades. A recent report [30] described a patient treated with six ultra-thin zirconia veneers from the maxillary right to left canine, with thicknesses ranging from 0.3 mm to 0.6 mm, which were able to fulfill the patient’s aesthetic demands, even after a 1-year follow-up. Another case series report [31] presented two patients treated with zirconia veneers, with one patient having restorations from the maxillary right lateral incisor to the left lateral incisor, and another patient from the maxillary right canine to the left lateral incisor. In both cases, thin zirconia veneers with a thickness of 0.6 mm were able to address the patients’ aesthetic concerns. Although these reports concluded that the translucent zirconia gave highly aesthetic results, the shade of the background teeth presented no stains, so there were no complications in matching the final color.

While companies that manufacture translucent zirconia claim that their products have excellent optical properties, and while the initial case reports display promising aesthetic results, limited information is available regarding the influence of different background shades, notably darker shades, on zirconia veneers with varying thicknesses. Therefore, this study aimed to evaluate the final shade of zirconia veneers with thicknesses of 0.5 mm, 0.75 mm, and 1.0 mm over teeth with A1, A2, A3, A3.5, and A4 shades and to test the null hypothesis that there is no difference in the shade for veneers with different thicknesses and background shades.

## 2. Materials and Methods

Three typodont (1560 Dentoform, Columbia Dentoform, Lancaster, PA, USA) maxillary right central incisor teeth were prepared for veneers with 1.0 mm incisal reduction and with facial reduction of 0.5 mm, 0.75 mm, and 1.0 mm. The three teeth were scanned with a chair-side CAD/CAM system (Emerald S Intraoral Scanner, Planmeca, Helsinki, Finland) and built-in software (PlanCAD Easy, Planmeca, Helsinki, Finland). A total of 75 veneer third-generation zirconia restorations (Katana UTML, Kuraray Noritake, Tokyo, Japan) were milled (n = 25 per thickness) with a dental laboratory milling machine (PrograMill PM7, Ivoclar Vivadent). Veneers were glazed (Cerabien ZR FC, Kuraray Noritake, Tokyo, Japan), sintered in a furnace (Programat S2, Ivoclar Vivadent), and polished (Zirconia Polisher Kerr Corporation, Brea, CA, USA), following the manufacturer’s recommendations.

Putty indexes (Splash Half-Time Set Putty Paks, Dent-Mat Holdings LLC, Lompoc) on the three prepared teeth were fabricated, and teeth were duplicated with resin composite (Filtek Supreme Flowable Restorative, 3M Oral Care, Saint Paul, MN, USA) with shades A1, A2, A3, A3.5, and A4. The veneers and background teeth with different shades were divided into the following groups, where each group had n = 25 shade evaluations:

- group 1 (0.5-A1), veneers with 0.5 mm thickness with background tooth shade A1;
- group 2 (0.5-A2), veneers with 0.5 mm thickness with background tooth shade A2;
- group 3 (0.5-A3), veneers with 0.5 mm thickness with background tooth shade A3;
- group 4 (0.5-A3.5), veneers with 0.5 mm thickness with background tooth shade A3.5;
- group 5 (0.5-A4), veneers with 0.5 mm thickness with background tooth shade A4;
- group 6 (0.75-A1), veneers with 0.75 mm thickness with background tooth shade A1;
- group 7 (0.75-A2), veneers with 0.75 mm thickness with background tooth shade A2;
- group 8 (0.75-A3), veneers with 0.75 mm thickness with background tooth shade A3;
- group 9 (0.75-A3.5), veneers with 0.75 mm thickness with background tooth shade A3.5;
- group 10 (0.75-A4), veneers with 0.75 mm thickness with background tooth shade A4;
- group 11 (1.0-A1), veneers with 1.0 mm thickness with background tooth shade A1;
- group 12 (1.0-A2), veneers with 1.0 mm thickness with background tooth shade A2;
- group 13 (1.0-A3), veneers with 1.0 mm thickness with background tooth shade A3;
- group 14 (1.0-A3.5), veneers with 1.0 mm thickness with background tooth shade A3.5; and
- group 15 (1.0-A4), veneers with 1.0 mm thickness with background tooth shade A4.

A color imaging spectrophotometer (Spectroshade Micro II, Oxnard, CA, USA) was used to obtain one frontal image of each restoration seated on each tooth with different shades. The contour of the facial surface was delineated following the borders of the restoration but held 1.0 mm away from the gingival aspect, as required by the software, to prevent pink gingiva color interference with the color measurement. The software provided a color shade map for each restoration, ranging from A1 to D4.

G-power calculation was used to determine the appropriate sample size for evaluating the ultra-translucent zirconia laminate veneers. The effect size was set to 0.25 (medium) or 0.5 (large), with an alpha of 0.05 and a power of 0.8. The evaluation involved three different thicknesses and five shades. The analysis showed that a total of 211 samples (for a medium effect size) or 58 samples (for a large effect size) were required. Furthermore, it was found that between 9.7 and 35.2 samples per group were needed, leading to a decision to use 25 samples per thickness and 15 samples per shade. Statistical analysis was conducted using a statistical software package (STATA, version 17—StataCorp, College Station, TX, USA). The analysis was conducted in three consecutive steps. First, the percentages of each of the output shades on the spectrophotometer shade map were calculated, by dividing the number of pixels of each particular shade over the total number of pixels of all shades that appeared on the shade map of each veneer image. The average percentage of each of the output shades was calculated for each of the fifteen groups, to obtain descriptive statistics. Second, a Kruskal–Wallis test was performed to assess the influence of veneer thickness and background shade on the output shade percentage distribution, followed by Dunn’s pair-wise comparison. The normality of the data was explored using the Shapiro–Wilk test. As the data were found to be non-normal, the Kruskal–Wallis test was deemed an appropriate statistical test for analysis. Finally, a multiple linear regression model was run, to simultaneously check the potential influence of both the veneer thickness and the background shade over the output shade distribution.

## 3. Results

**Figure 1:**
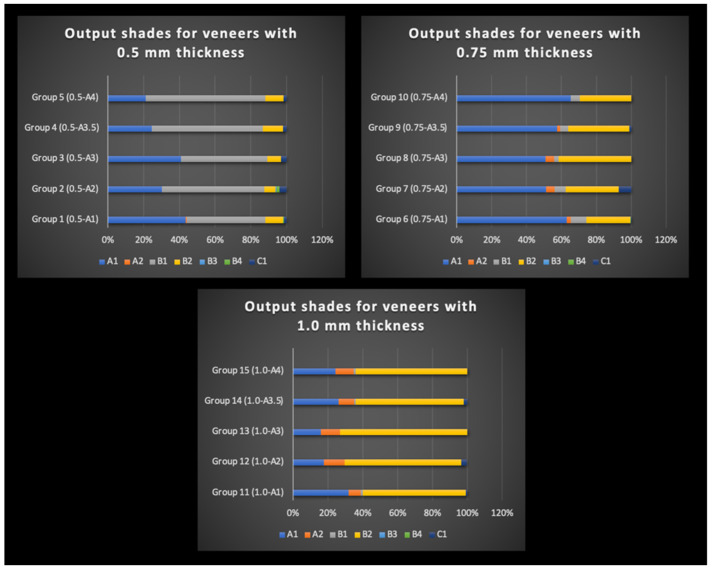
Output shade percentage distribution for zirconia veneers with 0.5 mm (**top left**), 0.75 mm (**top right**), and 1.0 mm (**bottom center**) thickness over backgrounds with shade A, A2, A3, A3.5, and A4.

The spectrophotometer analysis revealed variations in the output shade distribution in the shade map, with the final veneer shade varying depending on the thickness and background shade. Figure 1 presents the percentage distribution of veneer thicknesses (0.50 mm, 0.75 mm, and 1.00 mm) across the five background shades (A1, A2, A3, A3.5, and A4). Among the 0.50 mm thickness veneer groups, the highest percentage output shade was B1, followed by A1 shades, with a lower percentage distribution of the B2 shade. The results indicated that thinner veneers tended to exhibit a shade shift towards the B1 shade, while thicker veneers primarily showed the B2 shade.

**Figure 2:**
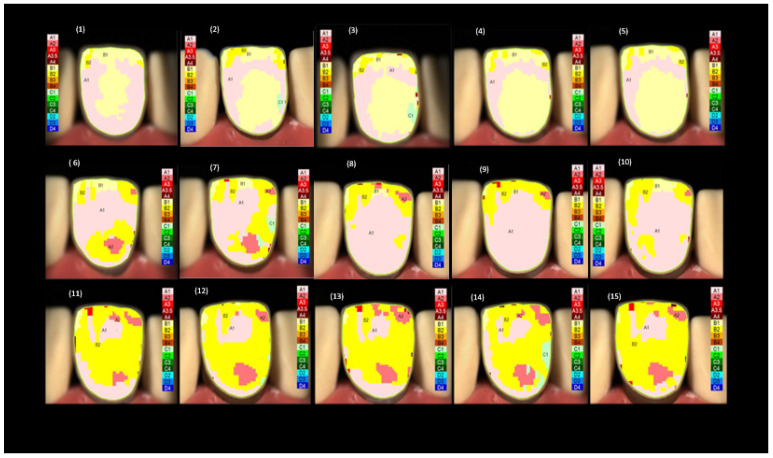
Representative spectrophotometer images displaying the shade maps of the 15 groups of the zirconia veneers with 0.5 mm, 0.75 mm, and 1.0 mm thickness over A1, A2, A3, A3.5, and A4 background shades.

In contrast, the shade maps for the 0.75 mm groups showed a greater percentage distribution of the A1 shade, followed by the B2 and B1 shades. The shade maps for the 1.00 mm thickness groups showed a higher percentage distribution of the B2 shade, followed by the A1 and A2 shades. Notably, the shade maps across all groups showed no or a low percentage distribution of the B3, B4, and C1 shades. To facilitate the analysis, the four main shades that appeared were the focus in the shade maps across all groups, namely A1, A2, B1, and B2. Examples of the shade maps for each of the three thickness groups are presented in Figure 2.

A Kruskal–Wallis test was run to accommodate the non-parametric data, to determine whether there was a statistically significant difference in the output shade distribution between each of the three veneer thicknesses groups. The same test was conducted to check the difference between the groups based on the background shades. Whenever the results were statistically significant, Dunn’s pair-wise comparison test with Bonferroni correction was run to determine which output shades showed a significant difference across the veneer thickness and the background shade groups.

The results in Table 1 show a statistically significant difference across all thickness groups for each of the output shades (n = 125 for each thickness group—25 samples over 5 background shades).

A pair-wise comparison Dunn test showed that all pair groups showed statistically significant differences for each of the main output shades appearing on the shade maps (A1, A2, B1, and B2). Comparing the output shade distribution across the five background shade groups (n = 75 samples each) showed a statistically significant difference across the five background shade groups for all the output shades, except B2. Pair-wise comparisons showed that the output shade A1 was significantly higher with the background shade A1 than all other background shade groups. The results comparing the output shades across the five background shade groups are summarized in Table 2.

To investigate the simultaneous influence of both veneer thickness and background shade on the shade map distribution, a multiple linear regression model was conducted considering the veneer thickness and the background shade as covariates and the percentage of the output shade as an outcome. The multiple linear regression analysis results are summarized in Table 3.

The results showed that, while adjusting for the background shade, increasing the veneer thickness from 0.5 mm to 0.75 mm resulted in a significant increase in the percentage of the output shades A1, A2, and B2 on the shade map, by 24.7%, 2.7%, and 22.6%, respectively (*p* < 0.001); and a significant decrease of the B1 shade by 48.8% (*p* < 0.001). When increasing the veneer thickness from 0.5 mm to 1.00 mm, there was a statistically significant increase in the percentage distribution of shades A2 and B2, by 9.8% and 55.5%, respectively, over the veneer shade map (*p* < 0.001), and a significant decrease in the percentage of output shades A1 and B1, by 8.7% and 54.8%, respectively (*p* < 0.001).

While adjusting for the veneer thickness, as compared to the A1 background shade, having darker background shades resulted in a significant decrease in the shade distribution of the output shade A1, by 13.8%, 10.8%, 10.6%, and 11.9% for the background shades (A2, A3, A3.5, and A4), respectively (*p* < 0.001). Darkening the background shades from A1 to A2 and A3 resulted in a statistically significant increase in the output shade A2 of around 2% for both background shades (*p* < 0.001). The percentage distribution of the B1 shade on the spectrophotometer shade map significantly increased, by 3.4% and 4.6%, with the background shades A2 and A3.5 compared to the A1 background shade (*p*-value = 0.015 and 0.001, respectively). Similarly, the percent distribution of the output shade B2 increased by 9% and 4.4% with the background shades A3 and A3.5 compared to the background shade A1 (*p*-value < 0.001 and *p* = 0.006, respectively). A final summary of the shades by percentage obtained in each group is shown in Table 4.

## 4. Discussion

Replicating natural teeth is challenging for clinicians working in the aesthetic area. Achieving optical, biological, and anatomical outcomes that mimic the natural dentition and satisfy patient demands is hindered by current restorative materials and color-matching techniques [32,33,34]. Color matching is a complex process that begins with selecting the appropriate tooth shade, which can be achieved through either visual assessment or with digital devices [35,36]. Traditionally, dentists have relied on prefabricated shade guides for visual assessment; however, this method is susceptible to various external factors that can compromise the accuracy of the outcome, such as the level of experience, eye fatigue, and lighting conditions [37].

Novel digital devices for shade selection have demonstrated improved reliability, by providing more consistent and accurate color values [38,39]. One such device is the spectrophotometer, which has been shown to produce accurate results for tooth color matching [40]. Some spectrophotometers divide the tooth or restoration into gingival thirds, middle third, and incisal third, and display the values on a black and white monitor, while more advanced devices provide a full shade map with a wide range of colors [41,42]. In our study, we utilized an advanced digital spectrophotometer (SpectroShade Micro II), which boasts a high-resolution LCD with a touch screen capable of handling 2 million image data points, for a complete mapping of the entire tooth or restoration for color assessment.

The shade A1 maxillary right central incisor veneers with thickness 0.5 mm, 0.75 mm, and 1.0 mm evaluated over background shades A1, A2, A3, A3.5, and A4 presented color alterations from the original A1. A summary of the shades by the percentage obtained in each group is shown in Table 4. The final shade displayed for veneers with 0.5 mm thickness was as follows: group 1 (0.5-A1) presented 44% B1, 43% A1, and 10% B2; group 2 (0.5-A2) displayed 57% B1, 30% A1, and 6% B2; group 3 (0.5-A3) presented 48% B1, 41% A1, and 8% B2; group 4 (0.5-A4) displayed 62% B1, 24% A1, and 11% B2. The final shade presented for veneers with a thickness of 0.75 mm was as follows: group 6 (0.75-A1) displayed 63% A1, 25% B2, and 9% B1; group 7 (0.75-A2) 51% A1, 42% B2, and 6% B1; group 8 (0.75-A3) presented 57% A1, 42% B2, and 3% B1; group 9 (0.75-A3.5) displayed 57% A1, 35% B2, and 5% B1; group 10 (0.75-A4) displayed 65% A1, 29% B2, and 5% B1. The final shade displayed for veneers with 1.0 mm thickness was as follows: group 11 (1.0-A1) presented 59% B2, 32% A1, and 7% A2; group 12 presented (1.0-A2) 67% B2, 18% A1, and 12% A2; group 13 (1.0-A3) displayed 73% B2, 16% A1, and 11% A2; group 14 (1.0-A3.5) presented 62% B2, 26% A1, and 9% A2; and group 15 (1.0-A4) displayed 64% B2, 24% A1, and 11% A2. Therefore, the null hypothesis that there was no difference in the shade for veneers with different thicknesses and background shades was partially rejected, because A1 veneers with thicknesses of 0.5 mm and 1.0 mm presented a higher percentage of shade B1 and B2, respectively.

The term "value" refers to the brightness of a color on a scale from white to black, where a high value is bright white, and a low value is dark gray. In the traditional tooth shade guide, the B1 shade represents the highest value, followed by A1 and B2. Our study found that the thinnest zirconia veneers, with a thickness of 0.5 mm, provided the highest percentage of B1 shade, which corresponds to a high value. Our findings are consistent with previous studies, demonstrating that thicker restorations generally result in lower values. For example, a recent study evaluated the color of ultra-translucent multilayered zirconia specimens with thicknesses of 0.5 mm and 0.7 mm over white and black backgrounds and found that thicker restorations had lower values [43]. Another recent study that examined monolithic zirconia disks with thicknesses of 0.5 mm, 1 mm, 1.5 mm, and 2 mm cemented with transparent and opaque types of cement also found that the material thickness affected the final color, with 0.5 mm specimens showing the highest value [44].

We used resin composite to standardize the background/stump shade across groups. However, the optical properties of resin composite and natural teeth differ based on how light interacts with the material through specular transmission, specular reflection, diffuse light reflection, and absorption and scattering [45]. Nevertheless, selecting natural teeth with standardized optical properties for comparison is challenging. Previous studies using natural teeth to evaluate veneer color matching noted the limitation of the potentially diverse color parameters and optical properties between different teeth [46,47,48]. Further research is necessary to determine how enamel optical properties, in particular, affect final shade matching. Furthermore, additional studies are also needed to evaluate the effect of thickness on other variables such as flexural forces [49] and hardness [50], to allow a more comprehensive overview of the tested ceramics.

This study aimed to guide clinicians in achieving the desired final shade for zirconia veneers with different thicknesses over different background shades. However, limitations were identified, such as the need for future studies on using color digital spectrophotometers to compare the final shade of zirconia veneers with different thicknesses using try-in resin cement in various shades. It would also be valuable to compare different zirconia brands using the same methodology and to conduct a study using final resin cement in different shades. Additionally, differences between the spectrophotometer and human vision were identified, but further studies are needed to evaluate the extent to which human vision agrees with the spectrophotometer findings, considering individual differences in color perception and the potential influence of lighting conditions. Lastly, studies comparing the spectrophotometer findings and visual assessment by clinicians could also provide valuable clinical data.

## 5. Conclusions

Based on the results of the color spectrophotometer evaluation conducted in this study, the final shade of zirconia laminate veneers with different thicknesses was found to be significantly influenced by the stump shade. The thinnest zirconia veneers, with 0.5 mm thickness, tended to display a higher value shade, B1, when placed over backgrounds A1, A2, A3, A3.5, and A4, whereas veneers with 0.75 mm and 1.00 mm thickness tended to display a lower value shade, B2.

These findings have important implications for clinicians seeking to obtain the desired final shade for zirconia dental veneers with different thicknesses. Future studies employing color digital spectrophotometers are warranted, to further evaluate the effect of try-in resin cement and different zirconia brands on the final shade of zirconia laminate veneers.

## Figures and Tables

**Table 1:** Distribution of the percentage of output shades according to veneer thickness. The table shows the results of the Kruskal–Wallis test for multiple comparisons of non-parametric data. 1: N = 125 samples (25 samples per veneer thickness*5 background shades) IQR: Interquartile range.

Output Shades	Veneer Thickness = 0.5 mm	Veneer Thickness = 0.75 mm	Veneer Thickness = 1.00 mm	*p*-Value
*A1, median (IQR)*	30.00 (25.00, 41.00)	59.00 (47.00, 69.00)	22.00 (17.00, 28.00)	<0.001
*A2, median (IQR)*	0.00 (0.00, 0.00)	0.00 (0.00, 6.00)	10.00 (8.00, 12.00)	<0.001
*B1, median (IQR)*	57.00 (48.00, 64.00)	6.00 (4.00, 8.00)	0.00 (0.00, 2.00)	<0.001
*B2, median (IQR)*	8.00 (6.00, 12.00)	28.00 (21.00, 40.00)	66.00 (61.00, 70.00)	<0.001
*B3, median (IQR)*	0.00 (0.00, 0.00)	0.00 (0.00, 0.00)	0.00 (0.00, 0.00)	0.002
*B4, median (IQR)*	0.00 (0.00, 0.00)	0.00 (0.00, 0.00)	0.00 (0.00, 0.00)	<0.001
*C1, median (IQR)*	0.00 (0.00, 4.00)	0.00 (0.00, 3.00)	0.00 (0.00, 0.00)	0.028

**Table 2:** Distribution of the % of output shades according to background shade. The table shows the results of the Kruskal–Wallis test for multiple comparisons of non-parametric data.

Output Shades	A1	A2	A3	A3.5	A4	*p*-Value
*A1, median (IQR)*	45.00 (30.00, 66.00)	30.00 (18.00, 44.00)	33.00 (18.00, 50.00)	30.00 (23.00, 41.00)	28.00 (20.00, 50.00)	<0.001
*A2, median (IQR)*	0.00 (0.00, 6.00)	7.00 (0.00, 10.00)	5.00 (0.00, 10.00)	0.00 (0.00, 8.00)	0.00 (0.00, 10.00)	0.010
*B1, median (IQR)*	7.00 (0.00, 44.00)	6.00 (0.00, 54.00)	3.00 (0.00, 41.00)	5.00 (2.00, 56.00)	10.00 (3.00, 63.00)	0.013
*B2, median (IQR)*	22.00 (9.00, 58.00)	34.00 (7.00, 63.00)	39.00 (11.00, 70.00)	31.00 (13.00, 58.00)	22.00 (12.00, 60.00)	0.220
*B3, median (IQR)*	0.00 (0.00, 0.00)	0.00 (0.00, 0.00)	0.00 (0.00, 0.00)	0.00 (0.00, 0.00)	0.00 (0.00, 0.00)	0.004
*B4, median (IQR)*	0.00 (0.00, 0.00)	0.00 (0.00, 0.00)	0.00 (0.00, 0.00)	0.00 (0.00, 0.00)	0.00 (0.00, 0.00)	<0.001
*C1, median (IQR)*	0.00 (0.00, 0.00)	3.00 (0.00, 7.00)	0.00 (0.00, 0.00)	0.00 (0.00, 4.00)	0.00 (0.00, 0.00)	<0.001

**Table 3:** Multiple linear regression analysis for percentage of output shades as outcome, considering both the veneer thickness and background shade as covariates.

Output Shade	Level	Coefficient	95% CI Lower	95% CI Upper	*p*-Value
A1					
*Veneer Thickness*	0.50 mm	Reference			
	0.75 mm	24.6619	21.6870	27.6369	<0.001
	1.00 mm	−8.6821	−11.657	−5.7071	<0.001
*Background Shade*	A1	Reference			
	A2	−13.8000	−17.6169	−9.9831	<0.001
	A3	−10.7916	−14.6215	−6.9617	<0.001
	A3.5	−10.6049	−14.4482	−6.7617	<0.001
	A4	−11.9333	−15.7503	−8.1164	<0.001
A2					
*Veneer Thickness*	0.50 mm	Reference			
	0.75 mm	2.6793	2.0575	3.3011	<0.001
	1.00 mm	9.8073	9.1855	10.4291	<0.001
*Background Shade*	A1	Reference			
	A2	2.173333	1.3755	2.9711	<0.001
	A3	2.043213	1.2427	2.8437	<0.001
	A3.5	0.224597	−0.5787	1.0279	0.583
	A4	0.253333	−0.5445	1.0511	0.533
B1					
*Veneer Thickness*	0.50 mm	Reference			
	0.75 mm	−48.8392	−50.9970	−46.6814	<0.001
	1.00 mm	−54.8312	−56.9890	−52.6734	<0.001
*Background Shade*	A1	Reference			
	A2	3.4400	0.6715	6.2085	0.015
	A3	−0.8845	−3.6624	1.8933	0.532
	A3.5	4.5765	1.7889	7.3641	0.001
	A4	8.5867	5.8182	11.3552	0
B2					
*Veneer Thickness*	0.5 mm	Reference			
	0.75 mm	22.6229	20.1951	25.0507	<0.001
	1.00 mm	55.5749	53.1471	58.0027	<0.001
*Background Shade*	A1	Reference			
	A2	3	−0.11491	6.1149	0.059
	A3	9.0195	5.8940	12.1449	<0.001
	A3.5	4.3956	1.2592	7.5320	0.006
	A4	1.5467	−1.5682	4.6616	0.329
B3					
Veneer Thickness	0.50 mm	Reference			
	0.75 mm	−0.1789	−0.2938	−0.0641	0.002
	1.00 mm	−0.1789	−0.2938	−0.0641	0.002
Background Shade	A1	Reference			
	A2	0.1867	0.03932	0.33401	0.013
	A3	−0.0517	−0.1996	0.0961	0.492
	A3.5	−0.0501	−0.1984	0.0983	0.507
	A4	−0.0533	−0.2007	0.0940	0.477
B4					
*Veneer Thickness*	0.50 mm	Reference			
	0.75 mm	−0.4314	−0.6481	−0.2147	<0.001
	1.00 mm	−0.4714	−0.6881	−0.2547	<0.001
*Background Shade*	A1	Reference			
	A2	0.3333	0.0553	0.6114	0.019
	A3	−0.2493	−0.5282	0.0297	0.080
	A3.5	−0.2451	−0.5250	0.0348	0.086
	A4	−0.2533	−0.5314	0.0247	0.074
C1					
*Veneer Thickness*	0.50 mm	Reference			
	0.75 mm	0.1345	−0.7897	1.0588	0.775
	1.00 mm	−1.0095	−1.9337	−0.0852	0.032
*Background Shade*	A1	Reference			
	A2	3.8933	2.7075	5.0792	<0.001
	A3	0.1769	−1.0130	1.3668	0.770
	A3.5	0.8655	−0.3285	2.0596	0.155
	A4	1.1333	−0.0525	2.3192	0.061

**Table 4:** Summary of the percentage distribution of output shades across different subgroups of background shades after adjusting for veneer thickness.

			Background Shades				
			*A1*	*A2*	*A3*	*A3.5*	*A4*
Zirconia Shade A1	*Veneers* *with 0.50 mm thickness*	Output Shades	Group 1 (0.5-A1)	Group 2 (0.5-A2)	Group 3 (0.5-A3)	Group 4 (0.5-A3.5)	Group 5 (0.5-A4)
		A1	43%	30%	41%	24%	21%
		A2	1%	0%	0%	0%	0%
		B1	44%	57%	48%	62%	67%
		B2	10%	6%	8%	11%	10%
		B3	0%	1%	0%	0%	0%
		B4	1%	2%	0%	0%	0%
		C1	1%	4%	3%	2%	2%
	*Veneers* *with 0.75 mm thickness*	Output Shades	Group 6 (0.75-A1)	Group 7 (0.75-A2)	Group 8 (0.75-A3)	Group 9 (0.75-A3.5)	Group 10 (0.75-A4)
		A1	63%	51%	51%	57%	65%
		A2	2%	5%	5%	2%	0%
		B1	9%	6%	3%	5%	5%
		B2	25%	30%	42%	35%	29%
		B3	0%	0%	0%	0%	0%
		B4	0%	0%	0%	0%	0%
		C1	0%	7%	0%	1%	0%
	*Veneers* *with 1.00 mm thickness*	Output Shades	Group 11 (1.0-A1)	Group 12 (1.0-A2)	Group 13 (1.0-A3)	Group 14 (1.0-A3.5)	Group 15 (1.0-A4)
		A1	32%	18%	16%	26%	24%
		A2	7%	12%	11%	9%	11%
		B1	1%	0%	0%	1%	1%
		B2	59%	67%	73%	62%	64%
		B3	0%	0%	0%	0%	0%
		B4	0%	0%	0%	0%	0%
		C1	1%	3%	0%	2%	0%

## Data Availability

Not applicable.
